# Endobiliary radiofrequency ablation for distal extrahepatic cholangiocarcinoma: A clinicopathological study

**DOI:** 10.1371/journal.pone.0206694

**Published:** 2018-11-15

**Authors:** Eui Joo Kim, Dong Hae Chung, Yoon Jae Kim, Yeon Suk Kim, Yeon Ho Park, Keon Kuk Kim, Jae Hee Cho

**Affiliations:** 1 Division of Gastroenterology, Department of Internal Medicine, Gil Medical Center, Gachon University College of Medicine, Incheon, Republic of Korea; 2 Department of Pathology, Gil Medical Center, Gachon University College of Medicine, Incheon, Republic of Korea; 3 Department of Surgery, Gil Medical Center, Gachon University College of Medicine, Incheon, Republic of Korea; Universitá Sapienza di Roma, ITALY

## Abstract

**Background:**

Most patients with distal extrahepatic cholangiocarcinoma have developed jaundice or cholangitis at the time of initial diagnosis, which can delay surgery. We aim to evaluate the actual EB-RFA ablation volume and validated the clinical feasibility of preoperative endobiliary radiofrequency ablation (EB-RFA) for resectable distal extrahepatic cholangiocarcinoma.

**Methods:**

The medical records of patients who underwent EB-RFA from July 2016 to June 2017 at a single tertiary academic medical center were reviewed. Inclusion criteria were patients with resectable distal extrahepatic cholangiocarcinoma who required preoperative biliary decompression. Clinical outcomes of EB-RFA were reviewed retrospectively and the surgical specimens were reevaluated.

**Results:**

Of the eight patients who required a delayed operation, preoperative EB-RFA was successfully performed without serious complications including peritonitis, hemobilia, or perforation. Although curative resection was attempted in all patients, one patient underwent open and closure due to hepatic metastasis. Seven patients underwent curative surgical resection and the histology revealed that median maximal ablation depth was 4.0 mm (range, 1–6) and median effective ablation length (histological ablation length/fluorosocopic ablation length) was 72.0% (range, 42.1–95.3).

**Conclusions:**

EB-RFA partially ablated human cancer tissue and preoperative EB-RFA might be a safe and feasible in patients with distal extrahepatic cholangiocarcinoma who require a delayed operation. Ablation of the target lesion longer than the estimated length by fluoroscopy may improve the efficacy of EB-RFA.

## Introduction

A malignant biliary tract obstruction (MBTO) can be caused by many etiologies, such as cholangiocarcinoma, pancreatic cancer, gallbladder cancer, or a metastatic lesion of non-biliary origin [1–3]. Jaundice and cholangitis are associated with high perioperative morbidity and mortality, but preoperative drainage may reverse the patholophysiological disturbance in patients waiting for surgery. Thus, preoperative biliary decompression is currently recommended in certain patients with jaundice and/or cholangitis [4].

The recently developed endobiliary radiofrequency ablation (EB-RFA) technique is increasingly performed in many countries for palliation of an MBTO [5–7]. Even improved survival of patients with pancreatic cancer has been reported [8, 9]. However, no studies have proven the feasibility and safety of EB-RFA as a bridge to surgery or as a neoadjuvant treatment for patients with MBTO caused by distal extrahepatic cholangiocarcinoma requiring preoperative biliary decompression.

Temperature-controlled RFA has been developed and used to treat cardiac arrhythmias and solid tumors, such as hepatocellular carcinoma [10, 11]. A temperature-controlled RFA catheter has an internal temperature sensor for monitoring tissue temperature, and the ablation can be terminated if the tissue temperature exceeds the preset target temperature, which could be helpful to control ablation depth and volume, and eventually avoid overheating and perforation. However, there is only limited evidence for temperature-controlled EB-RFA, including *in vivo* studies [12, 13].

In this study, we aim to elucidate the pathological tissue change and clinical feasibility of preoperative temperature-controlled EB-RFA for a homogenous group of patients with resectable distal extrahepatic cholangiocarcinoma, who required preoperative biliary decompression due to jaundice or cholangitis.

## Methods

### Patients

This was a single center, single arm, retrospective study. The medical records of patients who underwent EB-RFA for distal extrahepatic cholangiocarcinoma from June 2016 to August 2017 at a single tertiary academic medical center were reviewed retrospectively. The purpose of EB-RFA was decided based on whether there were any surgical plans before EB-RFA in the medical records, and the findings of the clinical imaging studies including computed tomography, magnetic resonance imaging, endoscopic ultrasound, endoscopic retrograde cholangiopancreatography (ERCP), or positron emission tomography at the initial diagnosis suggesting resectability. Target lesions were classified according to the growth pattern in imaging studies, which included expansive, periductal-infiltrating, and intraductal types [14]. Inclusion criteria were adult patients with resectable distal extrahepatic cholangiocarcinoma without evidence of distant metastasis, requirement of preoperative endobiliary decompression due to jaundice or cholangitis, and signed informed consent for EB-RFA. Exclusion criteria were MBTO other than the distal biliary tract, the MBTO does not require preoperative decompression, history of other malignancy, and EB-RFA for a palliative purpose. Gachon University Gil Medical Center, Institutional Review Board approved this study protocol (IRB No. GAIRB2017-262).

### Endobiliary radiofrequency ablation and radiological findings

**Fig 1:**
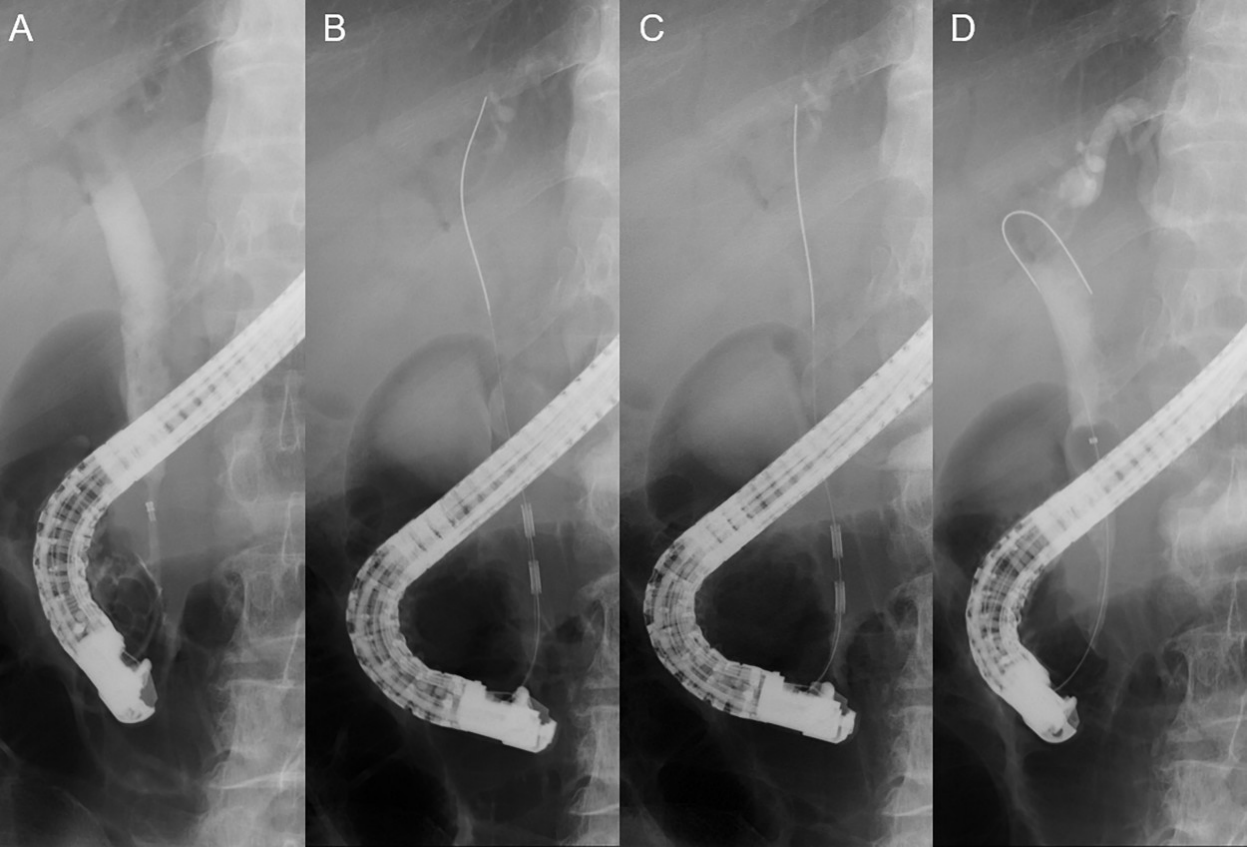
Endobiliary radiofrequency ablation (EB-RFA). A. Preprocedural cholangiogram showing distal extrahepatic bile duct stricture. B. EB-RFA with a temperature-controlled EB-RFA catheter at the proximal side of the stricture. C. Tandem overlapping EB-RFA at the distal side of the stricture. D. Endobiliary balloon sweeping removing necrotic tissues after successful EB-RFA.

Written informed consent was obtained for EB-RFA in all patients. A temperature-controlled EB-RFA catheter (ELRA^TM^, STARmed, Goyang, Korea) and RF generator system (VIVA combo, STARmed) were used for the procedure. A cholangiogram was acquired after successful bile duct cannulation using either a cannula or a papillotome. After passing the guidewire through the target lesion, the EB-RFA catheter was inserted along the guidewire. After positioning the EB-RFA catheter at the target lesion, 120 s of ablation (target temperature 80°C, 7–10 W) was performed. Tandem overlapping ablation was attempted to cover the entire length of the lesion if the lesion was fluoroscopically estimated to be longer than the EB-RFA probe. After removing the EB-RFA catheter, a balloon sweep was done to remove the intraluminal necrotic tissue. Then, an endoscopic retrograde biliary drainage (ERBD) stent was inserted as in conventional endoscopic decompression methods. The ERBD stent type was selected at the discretion of the endoscopist who had more than 5 years of therapeutic endobiliary procedure experience. Pre- and post-procedural diameters and the length of the stricture were measured fluoroscopically. Fluoroscopic ablation length, defined as the length from the distal electrode of the catheter to the leading point of ablation, was also measured with the ERCP image (Fig 1).

### Primary and secondary endpoints

The primary endpoint was the surgical feasibility of EB-RFA and technical difficulty, including immediate adverse events, such as perforation or massive hemobilia, and delayed adverse events including cholangitis, abscess formation, and severe adhesion, which can be obstacles for surgery. The secondary endpoints were histological ablation depth and length of EB-RFA, which can be used to predict effective EB-RFA volume. The difference in diameter of the bile duct stricture on a cholangiogram pre/post EB-RFA and the histological characteristics of the resected specimen including ablation depth and length were analyzed.

### Histological analysis

**Fig 2:**
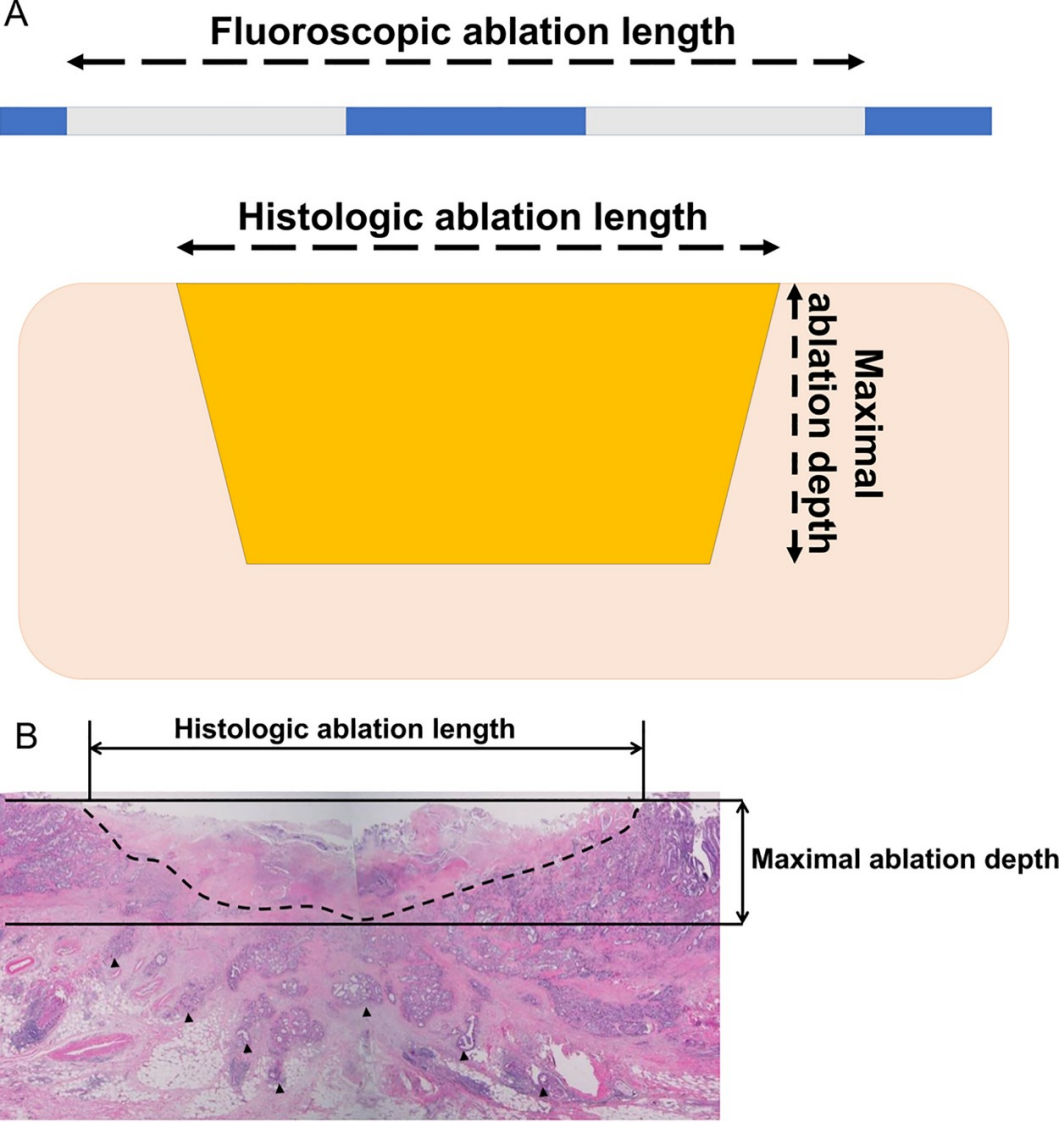
Histological analysis. A. Microscopic variables for the effective ablation. Measurement of fluoroscopic ablation length was based on the electrode marker and histological ablation length was based on the length of necrosis parallel to the bile duct in consecutive slides. Effective ablation was calculated as histological ablation length over fluoroscopic ablation length as a percentage. Maximum depth of ablation defined as necrosis between the basal lamina of the normal epithelium and the most deeply positioned necrotic inflammation. B. Microscopic image of the bile duct. Dotted line shows the margin of necrotic tissue and black arrow head shows malignant cell infiltration.

All surgical specimens and consecutive histological slides of resected specimens were reviewed by a pathologist, and the histological features of the target lesion were reported. Depth from the normal luminal surface to the lowest level of necrosis, effective ablation length, and periductal changes, including perforation, inflammation, and microabscess were evaluated. The maximal ablation depth was measured as the depth of necrosis defined as necrosis between the basal lamina of the normal epithelium and the most deeply positioned necrotic inflammation in the same manner as depth of tumor invasion for extrahepatic cholangiocarcinoma during T-staging according to the AJCC cancer staging system, 8th edition (Fig 2) [15]. Histological ablation length was measured as the length of necrosis parallel to the axis of the bile duct in consecutive slides (Fig 2). Effective ablation length was calculated as histological ablation length over the fluoroscopic ablation length multiplied by 100.

### Statistical analysis

As a single arm descriptive study, categorical variables are reported as numbers and percentages. Skewed data are reported as medians and ranges. Disease-free survival was estimated by the Kaplan–Meier method. All data were analyzed using SPSS software (ver. 23.0; IBM, Armonk, NY, USA).

## Results

### Baseline characteristics

Among the patients who underwent EB-RFA, eight were treated with preoperative EB-RFA (Table 1). All patients were diagnosed with extrahepatic cholangiocarcinoma confirmed by endoscopic biopsy and/or endoscopic ultrasound guided fine needle aspiration and underwent elective surgical resection. All patients received plastic endoscopic retrograde biliary drainage stents and none of the patients was treated with any other neoadjuvant treatment before surgery. Of the eight patients, hepatic metastasis was confirmed in one patient in a frozen biopsy during surgery after successful preoperative EB-RFA without any adverse events, and no further surgical management was required. Seven patients were included in the histological analysis.

**Table 1:** Demographics of included patients.

Characteristics	EB-RFA (N = 8)
Sex, male, n (%)	5 (62.5)
Age, median (range)	64.0 (54–75)
Distal extrahepatic cholangiocarcinoma, n (%)	8 (100)
Lab finding, median (range)	
At admission	
Hemoglobin, g/dL	13.2 (8.9–16.6)
WBC, /mm^3^	7880 (5580–10820)
CRP, mg/dL	2.5 (1.5–10.2)
Total bilirubin, mg/dL	15.5 (7.1–28.0)
At the time of surgery	
Hemoglobin, g/dL	11.3 (10.1–14.7)
WBC, /mm^3^	6060 (4340–14110)
CRP, mg/dL	0.64 (0.06–1.81)
Total bilirubin, mg/dL	2.3 (0.6–4.7)
Morphological growth type, n (%)	
Expansive / Periductal-infiltrating / Intraductal	6 (75.0) / 0 (0) / 2 (25.0)
EB-RFA power, n (%)	
7 W / 10 W	3 (37.5) / 5 (62.5)
Name of operation, n (%)^a^	
Whipple’s OP	7 (87.5)

^a^Hepatic metastasis was confirmed in one patient in a frozen biopsy during surgery after successful preoperative EB-RFA, only open and closure was done.

EB-RFA, endobiliary radiofrequency ablation; WBC, white blood cell; CRP, C-reactive protein; OP, operation

### Outcomes

All eight patients successfully underwent EB-RFA without any immediate adverse events, and the technical success rate was 100% (8/8). The median fluoroscopic improvement of diameters at the biliary stricture site were 1.3 (range: 0.7–1.7) mm. No evidence of perforation, hemobilia, cholangitis, cholecystitis, or death related to EB-RFA was detected in any patient (Table 2). Although one patient developed acute pancreatitis (abdominal pain with hyperamylasemia developed after EB-RFA), the pancreatitis was successfully managed with conservative management and the abdominal pain resolved within 48 hours after EB-RFA.

**Table 2:** Clinical outcomes following Whipple’s operation.

Variables	EB-RFA (N = 7)
R0 resection, n (%)	5 (71.4)
Surgical margin positive for malignancy, n (%)	2 (28.6)
TN stage (AJCC 7th / 8th), n (%)	
T3N0	3 (42.9) / 3 (42.9)
T3N1	4 (57.1) / 2 (28.6)
T3N2	NA^a^ / 2 (28.6)
EB-RFA related complications, n (%)	
Perforation	0 (0)
Hemobilia	0 (0)
Cholangits	0 (0)
Cholecystitis	0 (0)
Pancreatitis	1 (14.3)
Death	0 (0)
Interval between admission and OP, median (range), d	17 (10–57)
Interval between RFA and OP, median (range), d	8 (4–51)
Follow-up duration, median (range), d	222 (180–368)
Death after surgery, n (%)	1 (14.3)
30-days mortality	0 (0)
180-day overall survival rate, n (%)^b^	7 (100)
Other neoadjuvant treatment, n (%)	0 (0)
Adjuvant treatment, n (%)	6 (85.7)
Recurrence after surgery, n (%)	4 (57.1)
Estimated recurrence free survival, median (range), d	221 (163–279)

^a^N2 stage was not defined in the AJCC 7th Edition TNM Staging System

^b^The median survival was not reached by Kaplan-Meier method

**Fig 3:**
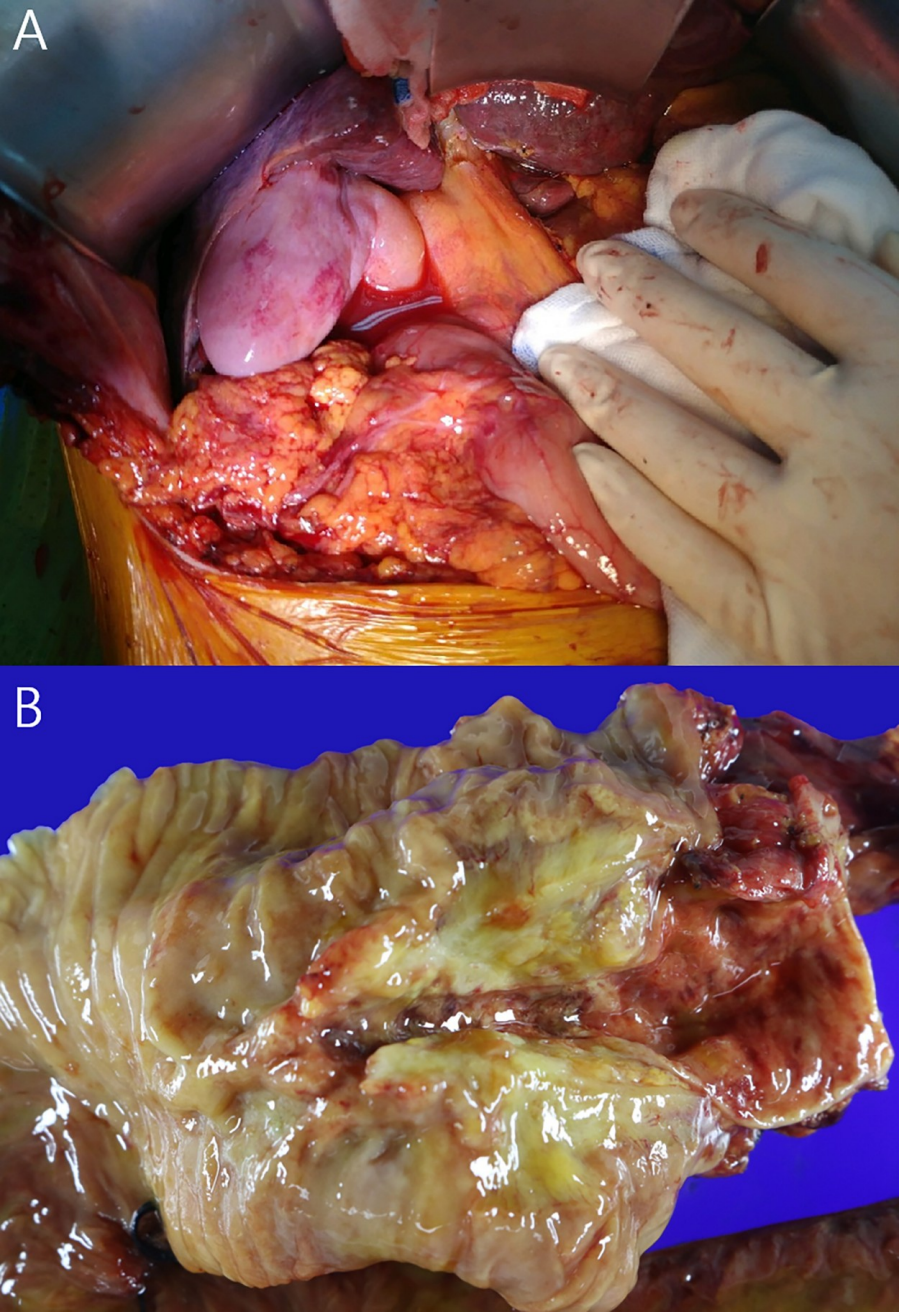
Surgical specimen. A. Intraoperative image of extrahepatic bile duct showing intact pericholedocal area. B. Macroscopic image of the bile duct necrosis showing yellowish color change.

Curative resection was attempted in 7 patients due to incidental finding of hepatic metastasis during surgery in one patient. Five patients underwent R0 resection and two patients had R1 resection with positive peripancreatic surgical margin; the R0 resection rate was 71.4% (5/7) (Table 2). In histological analyses, no microscopic perforations, vascular injuries, or pericholedochoal microabscesses formed. The median effective ablation length was 72.0% (range, 42.1–95.3%) and median maximal ablation depth was 4.0 (range, 1.0–6.0) mm (Table 3). No reported peritonitis, adhesion, or gross evidence of perforation was observed during the surgery (Fig 3). No 30-day mortality after EB-RFA or surgery was observed, and the 180-day overall survival rate was 100%. One death occurred during the follow-up period; the patient died 222 days after EB-RFA from gastrointestinal bleeding during adjuvant chemotherapy (S1 Dataset). Four patients developed new distant metastatic lesions during the follow-up period, and median disease-free survival was estimated to be 221 days (Table 2).

**Table 3:** Characteristics of the target lesion.

Characteristics	EB-RFA (N = 7)
Radiologic finding, median (range), mm	
Pre-procedural bile duct width	1.11 (0.57–1.44)
Post-EB-RFA bile duct width	2.35 (1.53–2.66)
Fluoroscopic stricture length	32.32 (26.23–36.90)
Fluoroscopic ablation length	33.74 (28.0–41.96)
Histologic finding, median (range), mm	
Maximal ablation depth	4.0 (1–6)
Histologic ablation length	21.0 (14–40)
Effective ablation length, median (range), %^a^	72.0 (42.1–95.3)

^a^Calculated as histologic ablation length over fluoroscopic ablation length

## Discussion

In this feasibility study, which included a homogenous group of patients with distal MBTO caused by distal extrahepatic cholangiocarcinoma, and who required biliary decompression before surgical treatment, the technical success rate of EB-RFA was 100% without immediate EB-RFA-related adverse events. No perforations, pericholedochal inflammation, or adhesions were observed. The R0 resection rate was 71.4% due to involvement of the microscopic surgical margin. However, it was comparable to that from other studies reporting R0 resection rates of 65.6–88.2% for mid to distal bile duct cancer [16, 17].

It is widely accepted that biliary decompression is required in certain patients with a resectable bile duct obstruction who are candidates for neoadjuvant treatment and in patients with cholangitis or severe jaundice [4]. Attempts to perform neoadjuvant treatment for patients with hilar cholangiocarcinoma have been reported, including radiotherapy or the recently developed photodynamic therapy during the delay period [18, 19]. However, there is insufficient evidence to support the consensus of local adjunctive treatment for MBTO caused by distal extrahepatic cholangiocarcinoma.

Some studies have reported the efficacy and safety of EB-RFA as a palliative treatment, and several *in vivo* studies have reported the ablation depth of EB-RFA in animals with normal bile ducts, there are insufficient data to estimate the ablation volume or depth in human subjects with a biliary malignancy [7, 9, 12, 20]. In one recent study, Atar et al. reported the result of *ex vivo* human bile duct radiofrequency ablation using bipolar impedance-controlled EB-RFA catheter [21]. The authors reported mean depth of necrosis (1.26–2.53 mm) in human *ex vivo* surgical specimens with normal bile ducts. However, because malignant biliary obstructive lesions can be eccentric, the contact area of the ablation catheter might not be constant throughout the procedure, and the ablation volume or depth might differ from those in animals or *ex vivo* surgical specimens with normal bile ducts. We analyzed ablation depth in human subjects with distal extrahepatic cholangiocarcinoma, and the results suggested a median ablation depth of 4.0 mm using a temperature-controlled RFA catheter. These results provide important fundamental data for further studies using temperature-controlled EB-RFA.

Recently, the cancer staging system is changed based on previous studies reporting that the invasion depth of malignant cells in “mm” is more effective to predict the prognosis of extrahepatic cholangiocarcinoma [15, 22]. In the 8^th^ edition of the AJCC cancer staging system, T-staging for extrahepatic cholangiocarcinoma is based on invasion depth of malignancy in “mm” and our histological data provide valuable information [23]. Because ablation depth in our sample suggested a median depth of 4.0 mm without any serious adverse events, the T1 (invasion depth < 5 mm from normal epithelium) stage is a good candidate indicator for EB-RFA, and good clinical outcomes can be expected in a subset of patients with extrahepatic cholangiocarcinoma. However, in our study, two patients underwent an R1 resection because the surgical peripancreatic margin (radial periductal margin) was positive for malignant cells microscopically and the median effective ablation length was 72.0%. Based on these results, the estimate of the target lesion with ERCP, which accompanies luminal imaging, may not be accurate enough to ablate the lesion completely by EB-RFA. In addition, the actual ablated length or volume was shorter or smaller than the ablation length or volume estimated by the length between catheter electrode markers. These results suggest that EB-RFA covering longer than the malignant stricture itself might improve the clinical efficacy of EB-RFA. Although the T1 stage according to the 8th edition of the AJCC cancer staging system is a good candidate indication for EB-RFA, limitations, including a poor estimate of the target lesion and ablation length as measured by fluoroscopic luminal imaging should be resolved in further studies.

Some limitations of this study should be discussed. Firstly, this study was a single-arm retrospective study with small number of patients and had lack of long-term follow-up data. Secondly, because there is concern about severe EB-RFA related adverse events such as hepatic infarction and hemobilia following EB-RFA for hilar bile duct which is a complex portion of the blood vessels structure, our study only included the distal extrahepatic cholangiocarcinoma [24, 25]. Thirdly, most of the patients had remnant cancer cells beyond the actual ablative field of EB-RFA due to the advanced T stage of the tumor. Lastly, because our study is the first to present the safety and feasibility of preoperative EB-RFA, there are not enough published data that support routine use of preoperative EB-RFA. Further studies to find the clinical efficacy of preoperative EB-RFA such as its effect on post-surgical risk for cancer recurrence are warranted.

In conclusion, EB-RFA with a temperature-controlled RFA catheter could result in coagulation necrosis of human cancer tissue. The median depth of ablation was 4.0 mm and the median effective ablation length was 72.0%. In addition, preoperative EB-RFA might be a safe and feasible adjunctive method with high technical success rates in patients with distal extrahepatic cholangiocarcinoma waiting for delayed surgery.

## Supporting information

S1 Dataset All relevant data available. (XLSX)
